# A Cinnamon-Derived Procyanidin Compound Displays Anti-HIV-1 Activity by Blocking Heparan Sulfate- and Co-Receptor- Binding Sites on gp120 and Reverses T Cell Exhaustion via Impeding Tim-3 and PD-1 Upregulation

**DOI:** 10.1371/journal.pone.0165386

**Published:** 2016-10-27

**Authors:** Bridgette Janine Connell, Sui-Yuan Chang, Ekambaranellore Prakash, Rahima Yousfi, Viswaraman Mohan, Wilfried Posch, Doris Wilflingseder, Christiane Moog, Eiichi N. Kodama, Pascal Clayette, Hugues Lortat-Jacob

**Affiliations:** 1 Institut de Biologie Structurale, UMR 5075, Univ. Grenoble Alpes, CNRS, CEA, F-38027 Grenoble, France; 2 School of Medical Technology, College of Medicine, National Taiwan University Hospital, Taipei, Taiwan; 3 Indus Biotech Pvt Ltd, Pune, India; 4 Laboratoire de Neurovirologie, Bertin Pharma, CEA, 92265 Fontenay aux Roses, France; 5 Division of Hygiene and Medical Microbiology, Innsbruck Medical University, Innsbruck, Austria; 6 INSERM U1110, Fédération de médecine translationnelle de Strasbourg (FMTS), Institut de Virologie, 3 rue Koeberlé, 67000 Strasbourg, France; 7 Division of Emerging Infectious Diseases, Miyagi Communitiy Health Promotion, Tohoku University School of Medicine, Bldg. 1, Rm. 515, 2–1 Seiryocho, Aoba-ku, Sendai 980–8575, Japan; George Mason University, UNITED STATES

## Abstract

Amongst the many strategies aiming at inhibiting HIV-1 infection, blocking viral entry has been recently recognized as a very promising approach. Using diverse *in vitro* models and a broad range of HIV-1 primary patient isolates, we report here that IND02, a type A procyanidin polyphenol extracted from cinnamon, that features trimeric and pentameric forms displays an anti-HIV-1 activity against CXCR4 and CCR5 viruses with 1–7 μM ED_50_ for the trimer. Competition experiments, using a surface plasmon resonance-based binding assay, revealed that IND02 inhibited envelope binding to CD4 and heparan sulphate (HS) as well as to an antibody (mAb 17b) directed against the gp120 co-receptor binding site with an IC_50_ in the low μM range. IND02 has thus the remarkable property of simultaneously blocking gp120 binding to its major host cell surface counterparts. Additionally, the IND02-trimer impeded up-regulation of the inhibitory receptors Tim-3 and PD-1 on CD4^+^ and CD8^+^ cells, thereby demonstrating its beneficial effect by limiting T cell exhaustion. Among naturally derived products significantly inhibiting HIV-1, the IND02-trimer is the first component demonstrating an entry inhibition property through binding to the viral envelope glycoprotein. These data suggest that cinnamon, a widely consumed spice, could represent a novel and promising candidate for a cost-effective, natural entry inhibitor for HIV-1 which can also down-modulate T cell exhaustion markers Tim-3 and PD-1.

## Introduction

Human Immunodeficiency Virus 1 (HIV-1) is the causative agent of Acquired Immunodeficiency Syndrome (AIDS), an immunosuppressive disease that creates vulnerability to lethal opportunistic infections and malignancies. More than 60 million people have been infected with HIV-1 since the beginning of the epidemic in 1981, half of which are now deceased. Due to the poor health systems and living conditions, developing countries are the hardest hit by this epidemic. Sub-Saharan Africa, is where the majority of new HIV-1 infections occur, these countries are home to 25.8 million people infected with HIV-1, accounting for almost 70% of the global HIV-1 infected population [1]. AIDS-related deaths continue to rise in Eastern Europe and Central Asia and the number of people infected with HIV-1 in India and Southeast Asia is also constantly increasing.

In this context, constant efforts are required to identify new anti-viral molecules that will be affordable to the majority of infected individuals, including those in emerging countries. For instance, naturally derived products potentially inhibiting HIV-1 are an advantageous strategy from multiple angles as they would not only be economically available to low and middle income countries where the epidemic is at its worst, but also potentially be suitable for oral ingestion. It is worth noting that several natural products have demonstrated anti-viral properties [2–6], including anti-HIV-1 activities [5]. Although, currently no plant-derived anti-HIV-1 drugs are used in clinical settings, promising data has been shown for certain natural candidates: (+)-Calanolide A, an anti-HIV-1 coumarin isolated from the fruit and twigs of the tree *Calophyllum lanigerum* var. *austrocoriaceum* (Guttiferae) which was described as a reverse transcriptase inhibitor [7]. Other products such as dicamphanoyl-khellactone (PA-334B) [8] inhibit primary, clinical as well as drug-resistant HIV-1 strains and Glycyrrhizic Acid (GL), a major bioactive tri-terpene glycoside of liquorice root, was shown to be a potent HIV-1 and HIV-2 inhibitor *in vitro* [9]. Of the many products potentially inhibiting HIV-1, only few have been found to target HIV-1 binding to the surface of CD4^+^ T cells [5]. Compounds such as Bezelladines A and B, obtained from the sponge Batzella sp., appeared to prevent CD4 binding to HIV-1 with IC_50_ of 10 and 25μM respectively, but their precise mechanism of action remains to be determined [10]. Cinnamon, a widely consumed spice, is commonly used in traditional medicine. Cinnamon extracts (*Cinnamomum cassia* bark and *Cardiospermum helicacabum* fruit) have already demonstrated a number of beneficial activities, including anti-inflammatory [11], anti-microbial, anti-oxidant, anti-tumor and immunomodulatory effects [12]. In particular cinnamon-derived procyanidin type A, which are condensed tannins, have shown anti-cancer properties, insulin-like action and inhibition of tau aggregation associated with Alzheimer’s disease [13–15]. Also anti-viral properties against both HIV-1 and HIV-2 were demonstrated using tannins [16, 17]. In an elegant study, mass spectrometric analyses of HIV-1 incubated with cinnamon, elderberry and green tea extract revealed virus-bound anti-viral compounds [18]. However little is known about their modes of action.

Here we investigated the potential of cinnamon extract as an anti-viral compound against HIV-1 and its ability to act early in the viral life cycle, targeting HIV-1 entry. Attachment to and entry of HIV-1 into host cells are crucial steps for HIV-1 infection and dissemination, therefore, representing an attractive target for anti-viral drugs, preventing infection from occurring in the first place [19]. Viral attachment and entry involves several cell surface molecules, which include heparan sulfate (HS), CD4 and co-receptors (CCR5/CXCR4), all recognized by gp120 [20–26], thus offering multiple sites for therapeutic intervention. We demonstrated that IND02, a cinnamon extract that we characterized as being composed of type A procyanidin trimer and pentamer polyphenols, inhibited the HIV-1 glycoprotein from interacting with cellular HS, CD4 and a monoclonal antibody (mAb 17b) used as a co-receptor surrogate. MAb 17b belongs to a group known as ‘‘anti-CD4i” antibodies, which recognizes a conserved element of gp120, induced by CD4 and partially overlapping the coreceptor binding site [27] and has been used in previous studies as a co-receptor surrogate [28, 29]. We observed that both IND02 and the purified IND02-trimer indeed bound to gp120 in the μM range, inhibiting CCR5- (R5-tropic) and CXCR4- (X4-tropic) utilizing gp120 from binding to mAb 17b and HS. Interestingly, we demonstrated that the IND02-trimer inhibited HIV-1 infection in multinuclear activation of the galactosidase indicator (MAGI) cells, with EC_50_ in the low μM range. The pentamer, which also inhibited the gp120-CD4 binding, displayed cytotoxicity preventing the evaluation of its anti-viral activity. We then further demonstrated the anti-viral capacity of the IND02-trimer in peripheral blood mononuclear cells (PBMCs) showing 1–7 μM ED_50_ against a range of clinical isolates from different clades. Additionally, the IND02-trimer down-modulated the negative immune regulators PD-1 (programmed death-1) and Tim-3 (T cell immunoglobulin mucin domain 3) on T cells, which are associated with T cell exhaustion during HIV-1 pathogenesis.

Although compounds having a single target and a well-defined mechanism of action are usually preferred for the development of drugs, it is worth noting that naturally derived and orally available molecules which have broad mechanisms of action are also clinically investigated. An extract from Spirulina, for example, has documented antioxidant, immunomodulatory, anti-inflammatory and antiviral activities [30].

The existing therapy against HIV-1, primarily targets the virus alone. However, for an efficient treatment, neutralization of the pathogen as well as host immune system modulation are equally important. Hence, molecules such as IND02-trimer act as a double-edged sword exerting its effects both on the virus and on restoring viral-induced defects in the host immune system. Therefore, we suggest that this molecule could serve as a novel and additional candidate for a cost-effective, natural entry inhibitor and immune modulator to complement the current available anti-HIV-1 inhibitors.

## Materials and Methods

### Materials

Biacore 3000, CM4 sensorchips, amine-coupling kit, and HBS-P buffer (10 mM HEPES, 150 mM NaCl and 0.005% (v/v) surfactant P20, pH 7.4) were obtained from GE Healthcare/Biacore (Uppsala, Sweden). The gp120 envelopes derived from two representative CXCR4- and CCR5-tropic strains, MN and YU2 respectively, were obtained from Immunodiagnostics (Massachusetts, USA). Soluble CD4 was obtained through the AIDS Research and Reference Reagent Program, Division of AIDS, NIAID, NIH and monoclonal antibody 17b was a kind gift from Dr. James Robinson. Streptavidin was obtained from Sigma Aldrich (Lyon, France).

### Preparation and characterization of IND02 and the IND02-trimer

The cinnamon-derived compound (IND02) was isolated as described (PCT Publication N°: 011/018793A1). Briefly, pulverized cinnamon (*Cinnamomum zylanicum*) bark was extracted with hydro alcohol (90%) at 25°C for 8 hrs in a stalked extractor. The resultant liquid was washed with ethyl acetate to remove any aromatic substances. The aqueous layer was passed through an adsorbent resin to trap all the polyphenols. The adsorbed components were eluted out over a gradient using an ethyl alcohol/water mixture. The eluents were monitored for active polyphenols using the thin layer chromatographic technique and the required fractions were combined and concentrated under a vacuum below 40°C followed by freeze-drying to remove traces of water to resolve the final powder product. This material was analyzed by HPLC, using a 250 mm x 4.6 mm Kromasil Reverse phase C-18 HPLC column 5μ pack and a JASCO model 2000 (PU2080, UV 2075, AS2055) HPLC system with CHROMPASS software. The extract was resolved with an isocratic mobile phase of 0.2% Orthophosphoric acid (aqueous): Acetonitrile (ratio 78:22) at 1.5 ml/min and monitored on-line by UV detection at 280 nm. The structures of the individual fractions were confirmed by mass and NMR spectroscopy.

The trimer form of the above composition of IND02 was isolated and purified in the following procedure. The crude cinnamon extract used for isolating the IND02 was used after removal of all unwanted compounds. The clarified extract was passed through a sequence of chromatographic columns consisting of a Nonpolar Adsorbent resin to trap the other oligomers and eluted with ethyl alcohol. The eluted solution containing the trimer was subjected to a flash chromatographic technique with Combiflash companion (Teledyne ISCO); using Redisep column (12g, Detection: 272 nm λ) and a gradient between solvent 1: 0.1% Aqueous formic acid and 2: 0.2% formic acid in Acetonitrile. The compound appearing at 19.5 mins in the HPLC was pooled and concentrated to get the trimer composition of IND02. The structure elucidation was carried out using NMR and LCMS techniques and showed a molecular mass of 864 g/mol and A type polyphenol linkage in the NMR analysis. The HPLC profile is comparable with the Cinnamatanin B1 Trimer Standard (ENZO life sciences).

### Surface Plasmon Resonance (SPR)-based binding platform

The interactions between gp120 and its ligands (CD4, mAb 17b and HS) were analyzed by Surface Plasmon Resonance (SPR) technology and using this platform [29], the inhibitory capacity of IND02 and the IND02-trimer was tested. For that purpose, CM4 sensorchips were activated with 50 μL of 0.2 M N-ethyl-N’-(diethylaminopropyl)-carbodiimide (EDC) and 0.05 M N-hydroxy-succimide (NHS) at 5 μL/min. Then soluble CD4 (10 μg/mL in 10 mM acetate buffer, pH 5), streptavidin (200 μg/mL in 10 mM acetate buffer, pH 4.2), mAb 17b (5 μg/mL in 10 mM acetate buffer, pH 5), MN gp120 (50 μg/ml in 5 mM maleate buffer, pH 6) or YU2 gp120 (50μg/mL in 10 mM acetate buffer, pH 4.8) were injected at 5 μL/min over one of the EDC/NHS activated flow cell until levels of 1200 (for sCD4), 700 (for mAb 17b), 3000 (for streptavidin), or 4500 (for gp120s) response units (RU) were achieved. HS (1 mM in phosphate-buffered saline) was biotinylated through its reducing end by reductive amination with 10 mM of biotin-LC-Hydrazine (Pierce) and captured on the streptavidin surface (20 μg/mL in 10 mM Hepes buffer pH 7.4 and 0.3 M NaCl) to a level of 60–80 RU as described [31]. Surfaces were then blocked with 1M ethanolamine (pH 8.5) for 5 minutes.

For binding studies, IND02 and IND02-trimer were diluted in HBS-P running buffer. Molecules under investigation (IND02, IND02-trimer alone or in complex with gp120) were injected over the different surfaces and the binding responses were recorded as a function of time. CD4, mAb 17b and gp120 surfaces were regenerated by 1 min injection of 10 mM HCl and the HS surface was regenerated with a 1 min injection of 0.05% SDS followed by a 3 min injection of 2 M NaCl.

### Determination of HIV-1 drug susceptibility

The sensitivity of infectious clones to IND02 and the IND02-trimer was determined in the multinuclear activation of the galactosidase indicator (MAGI) assay [32] with some modifications using viral preparations titrated as previously described [33]. Briefly, target cells (HeLa CD4/CCR5-LTR/β-gal; 10^4^/well) were plated in 96-well flat microtiter culture plates. On the following day, the medium was removed, and the cells were inoculated with HIV-1 NL4-3 clone or with the reference HIV-1-LAI strain (70 MAGI units/well, which gave 70 blue cells after 48 h of incubation) and cultured in the presence of various concentrations of the drug in fresh medium. The test drugs were added initially and viruses were immediately inoculated onto the cells. Forty-eight hours after viral exposure, all blue cells in each well were counted. The cytotoxicity of the compound was determined by the MTT method as previously described [34]. All experiments were performed in triplicate and AZT was used as reference anti-HIV-1 molecule. The 50% effective concentration (EC_50_ in μM) required to inhibit 50% of HIV-1 replication was calculated.

In order to confirm antiretroviral activity of the compounds, using a cell model closer to the pathophysiology, Peripheral Blood Mononuclear Cells (PBMCs) were separated from a buffy-coat from healthy HIV-, HCV-, HBV-seronegative blood donors by Ficoll-Hypaque density gradient centrifugation. The PBMCs were activated by incubation with 1 μg/ml phytohemagglutinin-P (PHA-P; Difco Laboratories) for three days in RPMI 1640 medium supplemented with 10% heat-inactivated fetal calf serum (FCS, +56 ◦C for 45 min), 2mM l-glutamine and a 1% antibiotic cocktail (penicillin, streptomycin, neomycin). After this mitogen activation, PBMCs were cultivated in the same culture medium supplemented with recombinant human interleukin-2 (rHuIL-2; 20 IU/mL) and treated and/or infected. PBMCs were infected either with the reference lymphotropic (-X4) HIV-1 LAI strain [35], the reference macrophage-tropic (-R5) HIV-1 BaL strain [36] or with 4 different clinical isolates (subtype A, B or C). All these viruses were amplified *in vitro* with PHA-P-activated blood mononuclear cells and 50% tissue culture infectious doses (TCID_50_) were calculated using Kärber’s formula [37], after limited-dilution titration. Viruses (125 TCID_50_) were incubated with a range of concentrations (0–20 μM) of the IND02-trimer molecule and added to PBMCs (m.o.i. ~ 0.001). Cell supernatants were collected at day 7 post-infection and stored at -20°C. Viral replication was measured by quantifying reverse transcriptase (RT) activity in the cell culture supernatants using the Lenti RT Activity Kit (Cavidi) and AZT was used as reference anti-HIV-1 molecule. EC_50_ were then calculated and expressed in μM.

### Flow cytometric analyses of IND02-trimer-treated infected PBMCs

PBMCs (1 x 10^6^/ml) were infected with 100 TCID50 of various HIV-1 strains (92UG037: primary R5-tropic strain; YU2B and BaL: R5-tropic lab strains, 93BR020: dual-tropic primary isolate; NL4-3: X4-tropic lab strain) and simultaneously treated with a range of concentrations of IND02-trimer in 24-well plates (Greiner) in duplicates. Cells were washed, fixed (4% paraformaldehyde) and stained on several days post infection (1-4-9-13-19) using the following mAbs against PD-1-Brilliant Violet 421, CD3-FITC, Tim-3-PE, CD4-PE-Cy7 (all from Biolegend) and CD8-APC (Miltenyi). Multicolor flow cytometric analyses were performed on a FACS Verse (BD Biosciences).

## Results

### Cinnamon extraction and characterization

**Fig 1:**
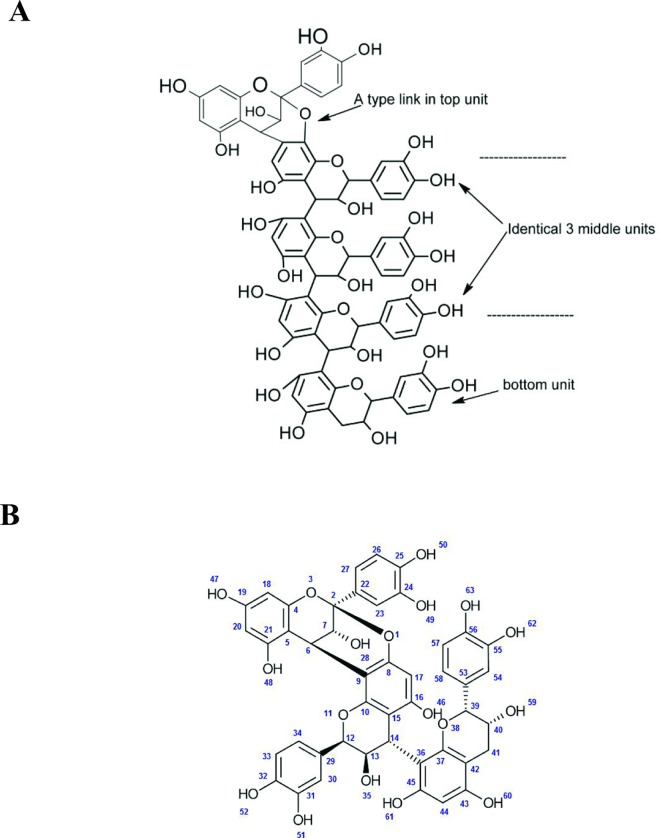
Structure of IND02. Structure of the IND02 compounds, isolated from cinnamon and characterized as pentameric (A) or trimeric (B) procyanidin A (see HPLC analyses in Figures A and B in S1 File).

Plant derived procyanidins are a class of condensed tannins possessing several monomeric units of catechins (+) and or epicatechins (-). Catechins, with a molecular mass of 288 g/mol, are the building blocks of flavonoid oligomers and are polyphenolic plant metabolites. Based on the type of linkage between the inter-monomeric units they are termed either as type A or type B. HPLC/MS analysis of the cinnamon extract (IND02) showed a minor peak (19.5 mins) and a major peak (38.4 mins) having molecular masses of 864 and 1440 g/mol respectively (Figures A and B in S1 File). This corresponds to a type A procyanidin trimer and pentamer polyphenols as shown by comparison with isolated standards of the trimer (Alexis Biochemicals ALX-350-365-M005, L-23448; RT: 19–20 mins) as well as pentamer (in-house standard, 95% pure; molecular mass 1440; EI-MS M-H ion peak at 1439; RT: 38.5 to 39.5 mins). The pentameric procyanidin A (which structure is shown in Fig 1A) represents 92% of the eluted material, while the trimeric structure (identical structure but with a single middle unit as shown in Fig 1B) represents 4% of the total material (Table A in S1 File). The trimer unit of the oligomer (molecular mass of 864 g/mol appearing at 19.5 mins in the above mentioned HPLC chromatogram) was also directly purified from cinnamon extract using nonpolar adsorbent chromatography followed by structure characterization using NMR and LC-MS techniques. By comparison of these data to that reported in the literature on cinnamtannin B1 [38] this establish the trimer A type poly phenol with a purity of 99% (Figure B and Table B in S1 File).

### IND02 and the IND02-trimer both bind to gp120

**Fig 2:**
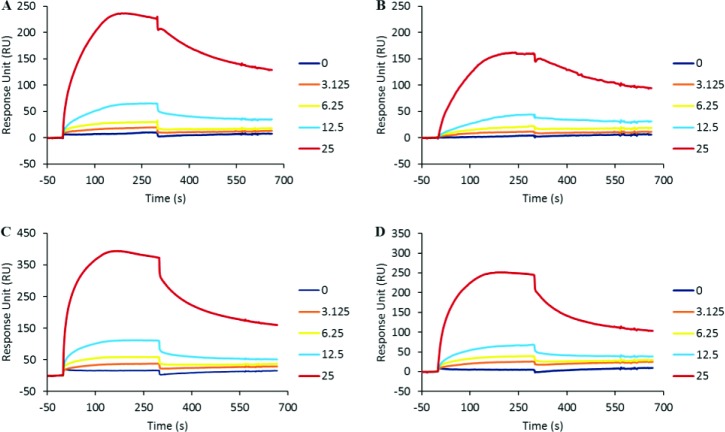
IND02 and IND02-trimer binding to gp120 immobilized sensorchip. IND02 (A and B) and IND02-trimer (C and D) at the indicated concentrations (in μM) was injected at 25 μl/min for 5 minutes over the immobilized gp120, and the specific binding response in resonance unit (RU) was recorded as a function of time (S). Overlay of the sensorgrams is depicted for IND02 and IND02-trimer over MN- (A and C) and YU2- gp120 (B and D) respectively. After each injection, the surface was regenerated by a short pulse of 10 mM NaOH.

In order to evaluate the potential anti-viral properties of cinnamon, we first underwent a series of biochemical binding experiments using surface plasmon resonance. Using various functionalized sensorchips developed to screen for HIV-1 entry inhibitors, we first assessed the capacity of the HIV-1 envelope glycoprotein to bind to IND02 and the IND02-trimer using a previously described assay [28]. Here, gp120 (either R5-tropic; YU2 or X4-tropic; MN) was immobilized onto a CM4 sensor chip and surface plasmon resonance (SPR) was used to measure changes in refractive index caused by the binding of the molecules injected in the fluid phase. Injection of IND02 or IND02-trimer over both MN and YU2 surfaces gave binding signals for a range of concentrations from 0 to 25 μM (Fig 2). This analysis revealed that IND02 and the IND02-trimer bound both X4- and R5-tropic gp120 similarly, suggesting a unique ability to interfere both with R5- and X4-tropic viral entry.

### IND02 and the IND02-trimer inhibit binding of gp120 to HS

**Fig 3:**
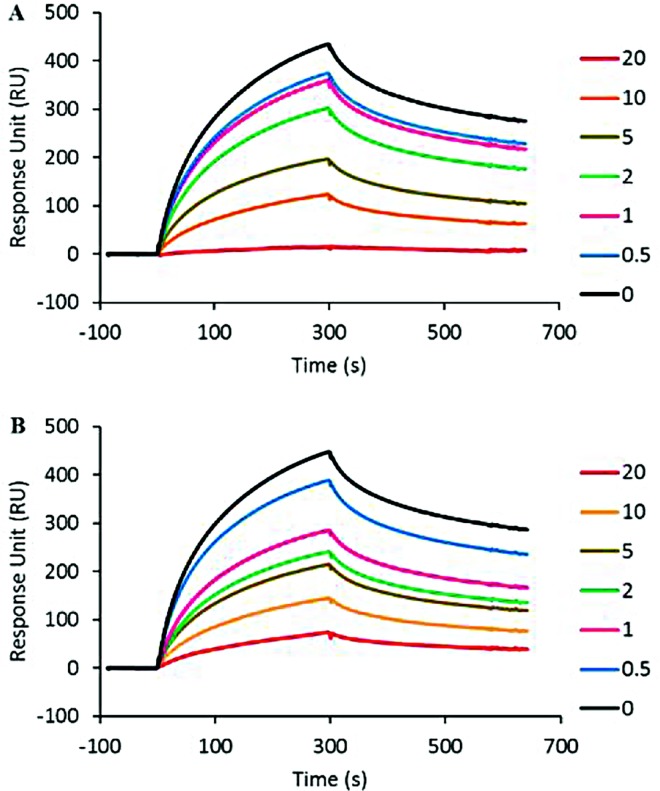
IND02 and IND02-trimer inhibition of gp120 binding to immobilized HS. HIV-1 gp120 (MN) was injected over the immobilized HS surface at 10 μl/min in the presence or absence of IND02 (A) or IND02-trimer (B) at the indicated concentrations (in μM). The specific binding responses in resonance unit (RU) was recorded as a function of time (s), and after each injection the surface was regenerated by a short pulse of 0.05% SDS and 2M NaCl.

Having established that the cinnamon-derived molecules bind to gp120, we aimed to investigate whether they inhibit gp120 from binding to cell-associated HS, a cell surface polysaccharide used by X4-tropic viruses in particular as an attachment molecule [19]. We performed a competitive surface plasmon resonance-based binding assay where the compounds of interest were analysed for their ability to inhibit binding of gp120 to HS. The R5-tropic YU2 envelope was not investigated in these experiments, as only X4-tropic gp120 strongly interacts with HS due to its highly positive charge of the V3 loop [39]. For that purpose, MN gp120 was injected at 50 nM over the HS surface, which gives rise to a strong binding signal (approx. 400 RU; Fig 3) indicating that gp120 strongly recognized the immobilized HS. Pre-incubation of gp120 with a range of concentrations of IND02 and IND02-trimer inhibited the binding in a dose-dependent fashion, yielding an IC_50_ of 7 ± 3.9 μM and 7.5 ± 3.5 respectively (Fig 3).

### IND02, but not IND02-trimer, inhibits binding of gp120 to CD4

**Fig 4:**
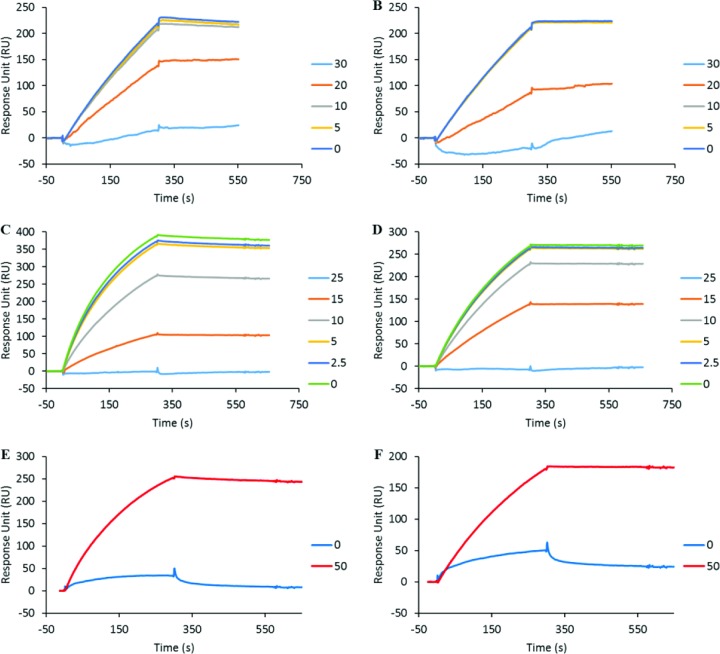
IND02 and IND02-trimer inhibition of gp120 binding to immobilized CD4 and mAb 17b. HIV-1 gp120 was injected over the immobilized CD4 at 10 μl/min in the presence or absence of IND02 at the indicated concentrations (in μM). The specific binding responses in resonance unit (RU) for MN- (A) and YU2- (B) gp120 were recorded as a function of time (s). Similarly gp120/CD4 complex (made by pre incubating gp120 and CD4 both at 25 mM for 30 minutes at room temperature) was injected over the immobilized mAb 17b surfaces at 10 μl/min in the presence or absence of IND02 (C,D) or IND02-trimer (E,F) at the indicated concentrations (in μM). Incubations with the inhibitor lasted at least 1 hour. The specific binding responses in resonance unit (RU) for MN- (C, E) and YU2- (D, F) gp120 were recorded as a function of time (s), and after each injection, the surface was regenerated by a short pulse of 10 mM HCl.

Next, we used a similar competition experiment where we injected the viral envelopes (either MN or YU2) over an immobilized CD4 surface in the presence of different concentrations of the cinnamon-derived inhibitors. These tests revealed that IND02 inhibits the YU2-CD4 interaction with an IC_50_ of 21.5 ± 3.5 μM and the MN-CD4 interaction with an IC_50_ of 20 ± 1.4 μM respectively (Fig 4A and 4B), in agreement with binding experiments of IND02 to X4 and R5 gp120. Using the same assay, the IND02-trimer did not show a significant inhibition of the viral envelopes binding to CD4 (data not shown). This suggests that the IND02 compound should bind to the CD4 binding domain, both for R5- and X4-tropic gp120.

### IND02 and the IND02-trimer block the co-receptor binding site of gp120

The region of gp120 that binds to CCR5 or CXCR4 is exposed and/or folded upon binding to CD4. It can be recognized by a number of monoclonal antibodies known as anti-CD4-induced (CD4i) epitope, which recognize gp120 residues induced by CD4 binding and have been used as co-receptor surrogates in a number of studies [27, 28, 40]. As this particular CD4-induced surface of the protein is critically involved in the entry mechanism [41, 42], we next investigated whether the cinnamon-derived compounds could also interfere with this domain. This was studied by analysing the ability of IND02 and the IND02-trimer to prevent gp120 binding to the anti-CD4i mAb 17b in the presence of soluble CD4. Complexes of gp120 and CD4 were incubated for at least 1 hour in the presence of the inhibitor and then injected over the mAb 17b surface. While gp120, in the absence of CD4 does not bind to the mAb 17b activated surface [29], the presence of CD4, induced a strong binding response (400 and 300 RU for MN and YU2 gp120 respectively; Fig 4C and 4D). A concentration-dependent inhibition was observed for IND02 with both X4- and R5-tropic envelopes interacting with mAb 17b displaying IC_50_ values of 8.5 ± 2.1 and 16 ± 1.4 μM respectively (Fig 4C and 4D) while for IND02-trimer, inhibition was observed from 50 μM only (Fig 4E and 4F).

### IND02 and the IND02-trimer display anti-HIV-1 activity

The antiviral activity of IND02 and the IND02-trimer was first investigated using a neutralization assay with MAGI cells. As shown in Table 1, the IND02-trimer exhibited inhibitory activity for both LAI and NL4-3 viruses, characterized by an EC_50_ of of 3.4 μg/mL (3.9 μM) and 2.8 μg/mL (3.2 μM) respectively. The similarity of the two results is consistent with the fact that both viruses bear the same envelope. The IND02-trimer did not show cytotoxicity at concentrations up to 100 μg/ml (115.7 μM). In contrast, 100 μg/ml of the pentamer (69.4 μM) showed cytotoxicity, preventing the evaluation of its potential anti-viral activity in the concentration range tested.

**Table 1:** Anti-viral activity of IND02 and AZT against HIV-1 LAI and NL4-3.

	EC_50_ (μM)		CC_50_ (μM)
	HIV-1 Lai	HIV-1_NL4-3_	
IND02 trimer	3.9 ± 1.6	3.2 ± 1.4	96 ± 4
IND pentamer	> 100	> 100	63 ± 9.7
AZT	0.012 ± 0.008	0.021 ± 0.011	> 100

The table shows the effective concentration, EC_50_ (mean of a triplicate determination ± SD in μM) of IND02-trimer or pentamer and AZT required to inhibit 50% of HIV-1 (strains LAI and NLA4-3) replication. The cytotoxic concentration, CC_50_ (mean of a triplicate determination ± SD in μM) of the IND02-trimer, pentamer and AZT that cause death to 50% of the viable cells is also shown in the table.

Next, we extended our investigations using PHA-P-activated peripheral blood mononuclear cells (PBMCs) and a panel of clinically relevant primary strains such as HIV-1 92UG029 (A-X4), HIV-1 92HT599 (B-X4), HIV-1 96USHIPS4 (B-X4/R5) and HIV-1 98IN017 (C-X4) in addition to HIV-1 –X4 and–R5 reference strains, HIV-1-LAI and HIV-1/Ba-L respectively. As shown in Table 2, the IND02-trimer displayed anti-viral activity, characterized by EC_50_ in the low μM range (0.8–7 μM) including a subtype C isolate which is endemic in the developing countries where the HIV-1 epidemic is at its worst. It is of interest to note that similar results were observed for the HIV-1-LAI strain between the two experimental models (EC_50_ of 3.9 and 4.6 μM for MAGI and PBMC, respectively).

**Table 2:** Anti-viral activity of the IND02-trimer and AZT against HIV-1 LAI, Ba-L and clinical isolates.

HIV strain (Clade-tropism)	LAI (B-X4)	Ba-L (B-R5)	92UG029 (A-X4)	92HT599 (B-X4)	96USHIPS4 (B-X4/R5)	98IN017 (C-X4)	CC_50_
IND02-trimer (μM)	4.6 ± 3	6.9 ± 2	2.3 ± 0	1.2 ± 1	2.3 ± 2	0.8 ± 0	23 ± 2
AZT (nM)	12 ± 3	15 ± 21	9 ± 4	12 ± 2	18 ± 10	6 ± 2	>1000

The table shows the effective concentration, EC_50_ (mean of a triplicate determination ± SD in μM) required to inhibit 50% of various HIV-1 stains replication incubated at 125 TCID_50_ with 150 000 PBMCs. The cytotoxic concentration, CC_50_ (mean of a triplicate determination ± SD in μM) of the IND02-trimer and AZT that cause death to 50% of the viable cells is also shown in the table.

### The IND02-trimer prevents up-modulation of Tim-3 and PD-1 on HIV-1-infected CD4^+^ and CD8^+^ T cells

**Fig 5:**
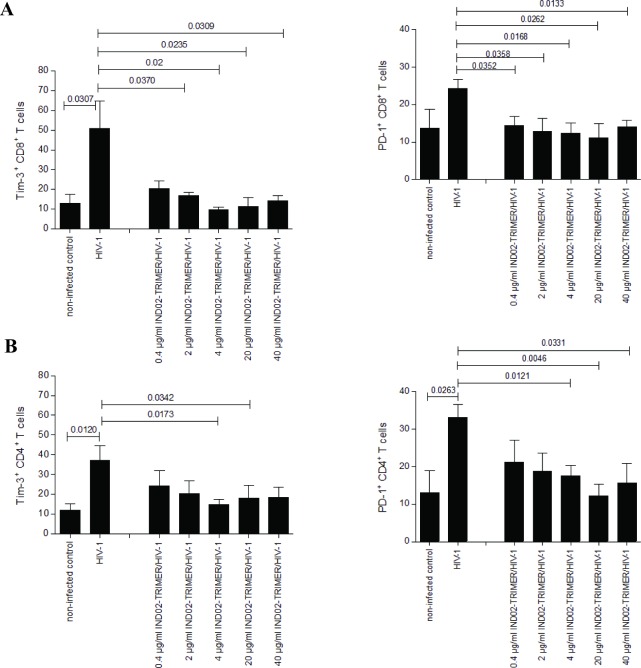
**Reduction of Tim-3 (left) and PD1 (right) expression on HIV-1 and IND02-trimer-treated CD8**^**+**^ **(A) and CD4**^**+**^ **(B) T cells.** Stimulated PBMCs were simultaneously incubated with 0.4 to 40 μg/ml (0.46–46.3 μM) IND02-trimer and HIV-1 (92UG037, BaL, YU2b, 93BR020 or NL4-3). Up-regulation of Tim-3 (left) and PD-1 (right) on CD8^+^ (A) and CD4^+^ (B) T cells was impeded by the IND02-trimer, as shown by multicolour FACS analyses of live T cells on day 9 post co-treatment. Experiments were repeated in 4–5 independent experiments with cells from different donors and statistical differences were analyzed by the GraphPad prism software using the unpaired student’s t-test (2-tailed).

Viral persistence is associated with increased expression of the inhibitory receptors Tim-3 and PD-1 on specific activated CD4^+^ and CD8^+^ T cells during HIV-1 and HCV infection. Therefore, we next studied the expression of these inhibitory receptors on the T cell subsets in long-term, HIV-1-infected PBMC cultures by multi-colour FACS analyses. We found that CD8^+^ T cells from HIV-1-infected PBMC cultures treated with 0.4 to 40 μg/ml (0.46–46.3 μM) IND02-trimer showed a significantly lower expression of Tim-3 on their surface than non-IND02-trimer -treated and infected control cells (Fig 5A, left). Also PD-1 expression on IND02-trimer-treated and HIV-1-exposed CD8^+^ T cells was significantly reduced compared to infected control cells at all concentrations tested (Fig 5A, right). The expression of PD-1 on CD8^+^ T cells was decreased to levels of non-infected control cells even at the lowest concentration of the compound tested (Fig 5A, right). Additionally, Tim-3 (Fig 5B, left) and PD-1 (Fig 5B, right) expression on CD4^+^ T cells were decreased on IND02-trimer treated and HIV-1-infected cells compared to infected control cells and were found to be slightly, but not significantly higher as on non-infected control cells. Measurements were performed on several days post treatment and infection, but to simplify matters, day 9 post infection (pI) from 4 to 5 independent experiments is depicted in Fig 5. Additionally, from day 13 pI onwards many dead cells were in the primary PBMC cultures especially in the HIV-1-only infected control, thereby impeding the gating strategy in some samples. Controls showed that Tim-3 and PD-1 were only slightly modulated on CD8^+^ or CD4^+^ cells alone (without addition of HIV-1) and were similar to non-infected control cells (Table 3). We here illustrated that the IND02-trimer reduced up-regulation of Tim-3 and PD-1 on CD4^+^ and CD8^+^ T cells in HIV-1-infected PBMCs.

**Table 3:** IND02-trimer alone did not alter expression of Tim-3 and PD-1 on CD8^+^ T cells.

IND02-trimer (μg/ml / μM)	% CD4^+^/PD-1^+^	% CD8^+^/PD-1
0 / 0	32.9	24.7
0.4 / 0.46	33.8	23.7
2 / 2.31	35.0	23.5
4 / 4.6	32.1	23.7
20 / 23.1	32.0	23.9
40 / 46	32.7	23.5

The table shows the level of expression (in %) of Tim-3 or PD-1 on CD8^+^ T cells alone on day 9 post exposure.

## Discussion

Plants have been an integral part of the ancient Indian, Chinese and Egyptian cultures for centuries and the importance of these natural remedies and medicines dates back as far as the Neanderthal period [43]. The benefits of the usage of naturally-derived medications are multi-faceted, from their ample abundance, no or low toxicity and their oral bioavailability. It is therefore evident that the resurgence of plant-based medications is seen by the numerous drugs in clinical trials, mainly for the treatment of cancer, immunological and central nervous system related diseases [44]. Of the ~27 million plant species that exist on our planet, only ~ 60 compounds are currently in the pipeline for the use as anti-cancer drugs [45] and several compounds have shown anti-viral properties against HIV-1.

Entry inhibition is an attractive target for antiretroviral action and offers prime sites for intervention [46]. Here we demonstrated in a biochemical analysis that a cinnamon extract (IND02 and IND02-trimer), derived from a mixture of type A procyanidin trimer and pentamer polyphenols binds to both R5- and X4-tropic gp120 and prevents gp120/Env binding to several of its cell-surface partners, including HS, CD4 (for IND02) and the co-receptor surrogate, mAb 17b. Although the gp120 domain to which IND02 and IND02-trimer bind has not been characterized, it is worth noting that the HS and co-receptor binding sites share a number of key residues, including the V3 loop base and the CD4 induced epitope [47, 48], to which the IND02 and IND02-trimer are thus likely to bind. As IND02, also inhibits the binding of gp120 to CD4, the pentameric form is thus likely to also overlap the CD4 binding site, which is spatially in close proximity to the V3 loop and the CD4i epitope.

Although a number of molecules targeting the CD4-binding domain have been described, very few compounds targeting the co-receptor binding site of gp120 have been reported. Most of them are antibodies, displaying only modest neutralizing activity, presumably because their access to the co-receptor binding domain is sterically and temporally restricted [49] during the pre-fusion steps, once the virus has been already bound by cell surface CD4. In this context, the low molecular masses 1440 g/mol and 864 g/mol (IND02 pentamer and trimer respectively) may allow these molecules to diffuse between the host cell membrane and the CD4-bound virus envelope, thereby displaying an ability to bind to the viral envelope as shown in the biochemical study. Altogether, these features bode well for the potential usage of this molecule as a dual-tropic entry inhibitor.

Based on the biochemical analyses of both the pentamer and trimer on the SPR platform, both molecular forms were tested in a MAGI assay where the pentameric IND02, but not the IND02-trimer displayed cytotoxicity. The trimer inhibited both HIV-1 NL4-3 and HIV-1 LAI strains with 50% effective doses around 3.2–3.9 μM. The trimers’ anti-HIV-1 activity was further confirmed on primary human PBMCs experimentally infected with a panel of primary HIV-1 strains (including a subtype C isolate, endemic in developing countries), thereby suggesting that this natural entry inhibitor should be further investigated. It could be noted that natural derived compounds [2, 3, 7, 8], peptidomimetic [50] or structure-based designed molecules [51] targeting the HIV-1 co-receptor binding site, as well as small drugs identified from high-throughput screens [52] also usually display anti-viral activity in the μM range. A possible development, for the IND02 compound, would be to conjugate it to nanoparticules or dendrimers as a way to enhance its efficacy and this will be investigated in future studies. In that context, it is interesting to note that a recently developed triazine derivative functionalized with aromatic amino acids was inactive in its monomeric form at 250 μM, showed limited anti-HIV-1 activity as a dimer (EC_50_ ~ 100 μM) but reached EC_50_ of 20 μM as a trimer [53].

Lectins and sulphated polysaccharides usually prevent the viral adsorption to cell surface features such as the glycocalix and DC-SIGN, but this binding is not sufficient to block the HIV-1 entry process. The inhibitory activity of the IND02-trimer, which also interferes with the co-receptor binding site, is therefore more relevant as an anti-HIV-1 compound. Another striking advantage of the IND02-trimer compared to polysaccharides, is that sulfated compounds usually target X4-, not R5-tropic viruses. The IND02-trimer might overcome this limitation by inhibiting both, R5- and X4-tropic virus strains.

Finally, we detected that IND02-trimer played a significant role in immune system regulation as it impeded up-regulation of the inhibitory receptors Tim-3 and PD-1 on CD8^+^ and CD4^+^ T cells in long-term HIV-1-infected PBMC cultures. Both molecules are associated with T cell exhaustion in chronic infection [54–58]. This suggests that the IND02-trimer may also exert an indirect beneficial effect by limiting T cell exhaustion, which would have to be further investigated.

These results place this natural compound in a competitive position compared to other molecules blocking HIV-1 infection at the gp120/Env-co-receptor interaction step and having additional beneficial effects on the immune system. These molecules include AMD 3100 (an X4 antagonist), which has been discontinued due to severe toxicity, and maraviroc (a CCR5 antagonist), which has been approved to be administered in combination with antiretroviral therapy, but only for patients infected with R5-tropic viruses. The use of maraviroc however could lead to the apparition//evolution of minority X4-tropic viruses, which has been correlated with a more rapid disease progression and there are already reported cases of viral resistance against maraviroc [59, 60]. It is also worth noting that such treatments are too expensive for developing countries and access to receive antiretroviral therapy is below 40% of the HIV-1-infected eligible population in low and middle income countries.

In conclusion, our study shows that the IND02-trimer, a natural compound, blocks HIV-1 replication. Although the inhibitory activity of the IND02-trimer required μM concentrations, the interest of this molecule lies in its ability to inhibit both X4- and R5-tropic HIV-1 and to target several conserved domains of the viral envelope glycoprotein. The IND02-trimer also appears to reduce exhaustion of the CD4^+^ T cell population by reducing Tim-3 and PD-1 expression, restoring normal T cell function. Extracted from cinnamon, a widely consumed spice, this low molecular weight compound can be produced on large scales and used in conditions compatible with the context of the countries where the epidemic is at its worst. Such cinnamon-derived compounds may be designed to be taken orally with other target specific anti-viral agents, and possibly interact with other anti-viral drugs, sometimes synergistically, additively or antagonistically. It is worth noting that IND02, comprising only type A procyanidin, is devoid of coumarin, a potentially toxic compound contained in substantial amount in cinnamon bark which has been shown to interfere with other drugs [61].

The IND02-trimer is usually isolated in kilogram quantities on a routine basis at the production facility and characterized using HPLC analysis to meet the requirement for commercial scale production. Therefore the methods to produce the IND02-trimer on a commercial scale are already well established. Stability studies on the IND02-trimer demonstrated that the compound is stable over 6 months in various formulations. The end cost of the IND02-trimer is also very well in the affordable range for a common man.

Further preclinical and clinical experiments are needed to evaluate the efficacy of the IND02-trimer *in vivo*. The IND02-trimer is water-soluble and therefore presumed to have better bioavailability than IND02. However, efforts are currently underway to enhance the bioavailability of the IND02-trimer through either enteric coating or by developing a cyclodextrin complex (unpublished data). Amongst the naturally-derived compounds with anti-HIV-1 activity, the IND02-trimer is the first of its kind to target the HIV-1 envelope and we believe that this entry inhibitor candidate molecule, which could be orally administered, deserves further investigation as a treatment option for HIV-1 infected patients.

## Supporting Information

S1 File **—Fig A. HPLC analysis of IND02.** The IND02, isolated from cinnamon extract, was analysed by HPLC, and followed by UV detection at 280 nm, demonstrating the presence of procyanidin trimer (~ 4% of the eluted material) and pentamer polyphenols (~ 92% of the eluted material). Black triangles indicate the beginnings and ends of each peak (see Table A). Unknown material (peak 1, 2, 4 and 5) represents 4.1% of the total UV trace.—**Fig B. HPLC analysis of IND02-trimer.** The sample (blue fraction on the chromatogram) was analyzed for Optical rotation, λmax in UV spectrum, and NMR spectroscopy on 600 MHz (Bruker Biospin Instrument). All the experiments were performed at 25°C, using 10 mg sample for ^1^H1 NMR and 50 mg sample for ^13^C NMR. The observed values were compared with literature reported values of cinnamtannin B1 (see Table B). The structure predicted using observed values matched with that of literature reported Cinnamatanin B1 [38, 62] and indicated the purity of the trimer to be 99%. This was also confirmed using Quantum Mechanics and Neuronal network calculations.—**Table A. Cinnamon-isolated IND02 characteristics**. The table shows the chromatographic characteristics of the cinnamon-isolated IND02. Peak numbers referred to Figure A.—**Table B. NMR analysis of IND02-trimer.** The table shows the comparison of the NMR output (600 MHz) for ^13^C and ^1^H in the observed experiment versus the reported literature, confirming the identity of the compound to be Cinnamatanin B1.
(PDF)
