# Supplementary material for: A Cinnamon-Derived Procyanidin Compound Displays Anti-HIV-1 Activity by Blocking Heparan Sulfate- and Co-Receptor- Binding Sites on gp120 and Reverses T Cell Exhaustion via Impeding Tim-3 and PD-1 Upregulation
Source: PLoS One. 2016 Oct 27;11(10):e0165386. doi: 10.1371/journal.pone.0165386 (PMC5082894; doi:10.1371/journal.pone.0165386)
Supplement: S1 File — —Fig A. HPLC analysis of IND02. The IND02, isolated from cinnamon extract, was analysed by HPLC, and followed by UV detection at 280 nm, demonstrating the presence of procyanidin trimer (~ 4% of the eluted material) and pentamer polyphenols (~ 92% of the eluted material). Black triangles indicate the beginnings and ends of each peak (see Table A). Unknown material (peak 1, 2, 4 and 5) represents 4.1% of the total UV trace.—Fig B. HPLC analysis of IND02-trimer. The sample (blue fraction on the chromatogram) was analyzed for Optical rotation, λmax in UV spectrum, and NMR spectroscopy on 600 MHz (Bruker Biospin Instrument). All the experiments were performed at 25°C, using 10 mg sample for 1H1 NMR and 50 mg sample for 13C NMR. The observed values were compared with literature reported values of cinnamtannin B1 (see Table B). The structure predicted using observed values matched with that of literature reported Cinnamatanin B1 [38, 62] and indicated the purity of the trimer to be 99%. This was also confirmed using Quantum Mechanics and Neuronal network calculations.—Table A. Cinnamon-isolated IND02 characteristics. The table shows the chromatographic characteristics of the cinnamon-isolated IND02. Peak numbers referred to Figure A.—Table B. NMR analysis of IND02-trimer. The table shows the comparison of the NMR output (600 MHz) for 13C and 1H in the observed experiment versus the reported literature, confirming the identity of the compound to be Cinnamatanin B1. (PDF) [file pone.0165386.s001.pdf]

**SUPPLEMENTARY INFORMATIONS**

# A cinnamon-derived procyanidin compound displays anti-HIV-1 activity by blocking heparan sulfate- and co-receptor- binding sites on gp120 and reverses T cell exhaustion via impeding Tim-3 and PD-1 upregulation

Bridgette Janine Connell[1], Sui-Yuan Chang[2], Ekambaranellore Prakash[3*], Rahima Yousfi[4], Viswaraman Mohan[3], Wilfried Posch[5], Doris Wilflingseder[5], Christiane Moog[6], Eiichi N. Kodama[7], Pascal Clayette[4] and Hugues Lortat-Jacob[1*]

**Figure A**

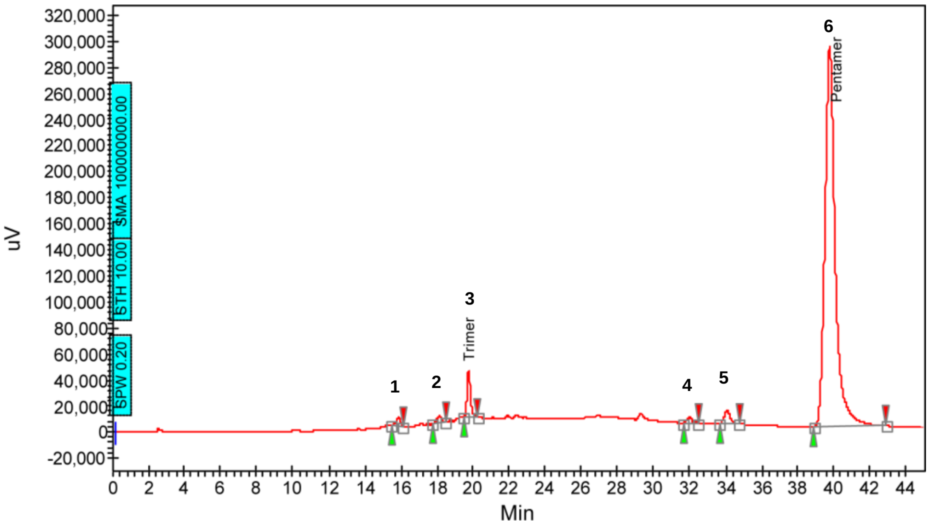


**Figure A: HPLC analysis of IND02.** The IND02, isolated from cinnamon extract, was analysed by HPLC, and followed by UV detection at 280 nm, demonstrating the presence of procyanidin trimer (~ 4 % of the eluted material) and pentamer polyphenols (~ 92 % of the eluted material). Black triangles indicate the beginnings and ends of each peak (see Table A). Unknown material (peak 1, 2, 4 and 5) represents 4.1 % of the total UV trace.

**Table A**

| **Peak N°** | **Identity** | **Start** (min) | **Time** (min) | **End** (min) | **Height** (uV) | **Area** (uV.Min) | **Area** (%) |
|---|---|---|---|---|---|---|---|
| **1** | unknown | 15.48 | 15.86 | 16.11 | 6623.4 | 1643.5 | 0.784 |
| **2** | unknown | 17.74 | 18.13 | 18.49 | 5099.6 | 1525.8 | 0.728 |
| **3** | Trimer | 19.43 | 19.72 | 20.24 | 38086.6 | 8250.9 | 3.937 |
| **4** | unknown | 31.65 | 31.98 | 32.53 | 4307.3 | 1415.4 | 0.675 |
| **5** | unknown | 33.66 | 34.07 | 34.78 | 10499.4 | 4075.1 | 1.945 |
| **6** | Pentamer | 38.92 | 39.82 | 42.93 | 293862.9 | 192653.9 | 91.931 |
| **Total** | | | | | 358479.1 | 209564.5 | 100.000 |

The table shows the chromatographic characteristics of the cinnamon-isolated IND02. Peak numbers referred to Figure A.

# Figure B

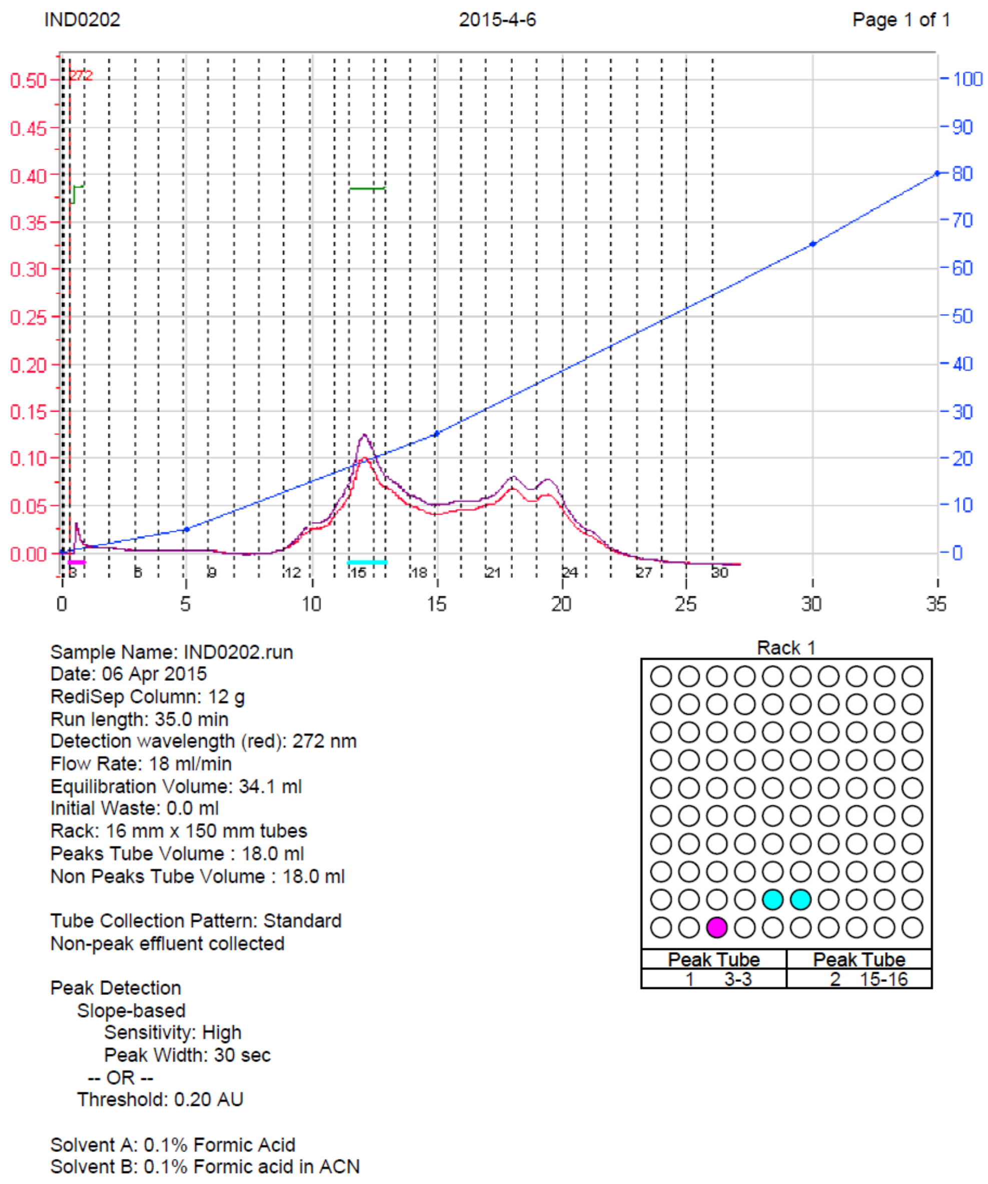


**Figure B: HPLC analysis of IND02-trimer.** The sample (blue fraction on the chromatogram) was analyzed for Optical rotation, λmax in UV spectrum, and NMR spectroscopy on 600 MHz (Bruker Biospin Instrument). All the experiments were performed at 25° C, using 10 mg sample for $^{1}$H1 NMR and 50 mg sample for $^{13}$C NMR. The observed values were compared with literature reported values of cinnamtannin B1 (Table B below). The structure predicted using observed values matched with that of literature reported Cinnamatanin B1 [1,2] and indicated the purity of the trimer to be 99%. This was also confirmed using Quantum Mechanics and Neuronal network calculations.

# Table B

| Ring | No | 13C | | 1H | |
|---|---|---|---|---|---|
| | | **Observed** | **Reported** | **Observed** | **Reported** |
| (upper unit) C | C-2 | 100.2 | 99.95 | | |
| | C-3 | 67.2 | 67.17 | 3.18 | 3.29 |
| | C-4 | 28.86 | 28.86 | 4.06 | 4.15 |
| A | C-5 | 154.2 | 156.74 | | |
| | C-6 | 98.3 | 98.33 | 5.87 | 5.97 |
| | C-7 | 157.9 | 157.8 | | |
| | C-8 | 96.6 | 96.6 | 5.92 | 6.02 |
| | C-9 | 154.2 | 154.14 | | |
| | C-10 | 105 | 104.9 | | |
| B | C -1’ | 132.5 | 132.44 | | |
| | C-2’ | 119.9 | 119.90 | 6.93 | 7.03 |
| | C-3’ | 145.56 | 145.5 | | |
| | C-4’ | 146.6 | 146.59 | | |
| | C-5’ | 115.8 | 116.18 | 6.75 | 6.84 |
| | C-6’ | 115.9 | 115.75 | 6.76 | 6.86 |
| Middle unit F | C-2 | 78.9 | 78.86 | 5.60 | 5.70 |
| | C-3 | 72.6 | 72.55 | 4.02 | 4.13 |
| | C-4 | 38.3 | 38.27 | 4.45 | 4.56 |
| D | C-5 | 155.8 | 155.76 | | |
| | C-6 | 96.1 | 96.09 | 5.70 | 5.80 |
| | C-7 | 151.1 | 151.08 | | |
| | C-8 | 106.8 | 106.42 | | |
| | C-9 | 151.8 | 151.78 | | |
| | C-10 | 106.8 | 106.73 | | |
| E | C-1’ | 131.8 | 131.76 | | |
| | C-2’ | 116.8 | 116.72 | 7.21 | 7.32 |
| | C-3’ | 145.9 | 145.89 | | |
| | C-4’ | 146.3 | 146.27 | | |
| | C-5’ | 116.2 | 115.79 | 6.73 | 6.82 |
| | C-6’ | 121.4 | 121.36 | 7.10 | 7.19 |
| Lower unit | C-2 | 80.4 | 80.27 | 4.28 | 4.38 |
| I | C-3 | 67.6 | 65.71 | 3.76 | 3.85 |
| | C-4 | 29.9 | 29.84 | 2.74 | 2.83 |
| G | C-5 | 155.6 | 155.99 | | |
| | C-6 | 96.5 | 96.51 | 6.01 | 6.10 |
| | C-7 | 155.6 | 155.53 | | |
| | C-8 | 108.9 | 108.85 | | |
| | C-9 | 156.1 | 155.78 | | |
| | C-10 | 100.1 | 100.08 | | |
| H | C-1’ | 132.2 | 133.17 | | |
| | C-2’ | 115.5 | 115.48 | 6.72 | 6.82 |
| | C-3’ | 145.4 | 145.31 | | |
| | C-4’ | 145.8 | 145.74 | | |
| | C-5’ | 116 | 116.03 | 6.64 | 6.76 |
| | C-6’ | 119.5 | 119.45 | 6.63 | 6.72 |

The table shows the comparison of the NMR output (600 MHz) for $^{13}C$ and $^{1}H$ in the observed experiment versus the reported literature, confirming the identity of the compound to be Cinnamatanin B1.

**Note:**

1. While prediction methodology relied on standard solvent chemical shift to predict the chemical shifts, the observed chemical shifts were referenced to TMS [$(CH_3)_4Si$], the standard deviation between the predicted and the observed chemical shifts is 0.095 ppm.
2. IND02-trimer has fourteen -OH groups and all of them appear at 4.52 ppm as a broad peak. They are not listed in the above table.

**Supplementary reference**


1. Ben Amor N, Bouaziz A, Romera-Castillo C, Salido S, Linares-Palomino PJ, et al. (2007) Characterization of the intracellular mechanisms involved in the antiaggregant properties of cinnamtannin B-1 from bay wood in human platelets. J Med Chem 50: 3937-3944.
2. Kamiya K, Watanabe C, Endang H, Umar M, Satake T (2001) Studies on the constituents of bark of Parameria laevigata Moldenke. Chem Pharm Bull (Tokyo) 49: 551-557.
